# Regulation of hTERT by BCR-ABL at multiple levels in K562 cells

**DOI:** 10.1186/1471-2407-11-512

**Published:** 2011-12-09

**Authors:** Juin Hsien Chai, Yong Zhang, Wei Han Tan, Wee Joo Chng, Baojie Li, Xueying Wang

**Affiliations:** 1 Department of Biochemistry, Yong Loo Lin School of Medicine, National University of Singapore, 8 Medical Drive, 117597 Singapore, Singapore; 2 Department of Biochemistry, Yong Loo Lin School of Medicine, Cancer Science Institute of Singapore (CSI), National University of Singapore, Singapore, Singapore; 3 Department of Medicine, Yong Loo Lin School of Medicine, National University of Singapore, Singapore, Singapore; 4 Department of Haematology-Oncology, National University Cancer Institute of Singapore, National University Health System, Singapore, Singapore; 5 Bio-X Center, Key Laboratory for the Genetics of Developmental and Neuropsychiatric Disorders, Ministry of Education, Shanghai Jiao Tong University, Shanghai, People's Republic of China

## Abstract

**Background:**

The cytogenetic characteristic of Chronic Myeloid Leukemia (CML) is the formation of the Philadelphia chromosome gene product, BCR-ABL. Given that BCR-ABL is the specific target of Gleevec in CML treatment, we investigated the regulation of the catalytic component of telomerase, hTERT, by BCR-ABL at multiple levels in K562 cells.

**Methods:**

Molecular techniques such as over expression, knockdown, real-time PCR, immunoprecipitation, western blotting, reporter assay, confocal microscopy, telomerase assays and microarray were used to suggest that hTERT expression and activity is modulated by BCR-ABL at multiple levels.

**Results:**

Our results suggest that BCR-ABL plays an important role in regulating hTERT in K562 (BCR-ABL positive human leukemia) cells. When Gleevec inhibited the tyrosine kinase activity of BCR-ABL, phosphorylation of hTERT was downregulated, therefore suggesting a positive correlation between BCR-ABL and hTERT. Gleevec treatment inhibited *hTERT *at mRNA level and significantly reduced telomerase activity (TA) in K562 cells, but not in HL60 or Jurkat cells (BCR-ABL negative cells). We also demonstrated that the transcription factor STAT5a plays a critical role in *hTERT *gene regulation in K562 cells. Knockdown of STAT5a, but not STAT5b, resulted in a marked downregulation of *hTERT *mRNA level, TA and hTERT protein level in K562 cells. Furthermore, translocation of hTERT from nucleoli to nucleoplasm was observed in K562 cells induced by Gleevec.

**Conclusions:**

Our data reveal that BCR-ABL can regulate TA at multiple levels, including transcription, post-translational level, and proper localization. Thus, suppression of cell growth and induction of apoptosis by Gleevec treatment may be partially due to TA inhibition. Additionally, we have identified STAT5a as critical mediator of the *hTERT *gene expression in BCR-ABL positive CML cells, suggesting that targeting STAT5a may be a promising therapeutic strategy for BCR-ABL positive CML patients.

## Background

Chronic myeloid leukemia (CML) was the first human cancer to be linked to a consistent chromosomal abnormality [1]. The cytogenetic characteristic of CML is the formation of the Philadelphia chromosome (Ph), by the translocation of chromosome 22 and chromosome 9. As a result, part of the *breakpoint cluster region *(*BCR*) gene from chromosome 22 fuses with the *ABL *gene on chromosome 9. Transcription of this fusion gene results in constitutively active p210 or p190 BCR-ABL tyrosine kinase [2], which is detected in 95% of CML and in 20-30% of adult acute lymphoblastic leukemia (ALL), respectively [3,4]. BCR-ABL has a higher tyrosine kinase activity than its cellular counterpart, c-ABL [5]. The deregulated activity of BCR-ABL leads to uncontrolled cell proliferation and reduced apoptosis [6]. BCR-ABL is predominantly localized in the cytoplasm where it interacts with various cellular proteins. These proteins are either phosphorylated by BCR-ABL or promote phosphorylation of their interaction partners, which in turn triggers the activation of numerous signaling pathways, including RAS-RAF, MAPK, PI-3-Kinase, c-JUN and c-MYC pathways [7-10].

As the tyrosine kinase activity of BCR-ABL is essential for its transforming ability [11], specific targeting of the BCR-ABL tyrosine kinase provides a promising strategy for CML therapy. Gleevec (Imatinib mesylate or STI571), a tyrosine kinase inhibitor which has revolutionized CML therapy, is the current gold standard treatment for CML. Gleevec possesses specificity for Abl, BCR-ABL, c-Kit and the PDGF receptor. It competitively binds to the ATP-binding site of BCR-ABL and prevents a conformational switch to the oncoprotein's active form. This inhibits BCR-ABL activation through autophosphorylation, and blocks its downstream signal transduction [12]. About 96% of CML patients exhibited complete hematologic responses (CHR) and major cytogenetic responses (MCR) to Gleevec treatment, and approximately 55% of ALL patients showed positive responses to Gleevec treatment [13,14].

Human telomerase is a ribonucleoprotein complex consisting of two core components, telomerase reverse transcriptase (human TERT, hTERT) and telomerase RNA template (human TER, hTER). TERT is a class of enzyme that creates single-stranded DNA using single-stranded RNA as a template, whilst TER serves as a template for addition of telomeric repeats (TTAGGG) to DNA strands. By using TER, TERT can cap and protect chromosome ends by adding a six-nucleotide repeating sequence, 5'-TTAGGG (in all vertebrates, the sequence differs in other organisms) to the 3' strand of chromosomes [15]. The expression of hTERT is the rate-limiting determinant of human telomerase activity (TA) and is thought to be a sensitive indicator of telomerase function and activity. However, the means by which TA is regulated remain largely unknown. TA has been observed in ~85% of all human tumors, suggesting that the immortality conferred by telomerase plays a key role in malignant transformation [16]. TA has been shown to increase in the bone marrow cells of patients with CML during disease progression [17].

Transfection of the catalytic subunit of telomerase, hTERT, into cultured human primary cells transformed with SV40 large T antigen and N-ras oncogene allows cells to overcome crisis and ultimately achieve malignancy. This suggests that telomerase upregulation may contribute actively to cellular immortalization and tumorigenesis, in human cells [18]. Therefore, telomerase can be considered as an attractive target for cancer diagnosis and anticancer therapy. TA and the expression of telomerase components are regulated at multiple levels, including transcription and post-transcription, accurate assembly, and proper localization [19]. TA can also be regulated at the post-translational level as studies have shown that protein kinase C (PKC) α and AKT/protein kinase B (PKB) can upregulate human TA through phosphorylation of hTERT [20,21].

Several studies have reported that Gleevec can regulate TA [22-25]. However, the mechanism by which Gleevec affects TA in BCR-ABL-expressing cells is unclear. Contradicting results were obtained from different studies; some have shown that Gleevec treatment could increase TA and telomere length [26,27], while a more recent study indicated that Gleevec reduced TA in K562, BCR-ABL positive cells [25].

Given that BCR-ABL is the specific target of Gleevec, we surmised that Gleevec affects TA by regulating the expression and activation of telomerase via BCR-ABL. In this study, we investigated the effects of Gleevec on TA in a BCR-ABL positive cell line (K562) and deficient cell lines (HL60 and Jurkat). Our results indicated that Gleevec treatment dramatically inhibits TA and decreases hTERT expression at the mRNA level in K562, but not in HL60 and Jurkat cells. Moreover, knocking down of STAT5a by siRNA resulted in a marked downregulation of *hTERT *mRNA level, protein level, and TA in K562 cells. We also found that K562 cells exhibit a significant increase in hTERT phosphorylation at tyrosine, which was reduced upon Gleevec treatment in K562 cells, but absent in HL60 cells. Furthermore, we also observed the release of hTERT from the nucleoli to the nucleoplasm of Gleevec-treated K562 cells. These results highlight the potential role of BCR-ABL in telomerase regulation and imply that BCR-ABL might regulate telomerase expression and activity at the transcriptional level via the JAK-STAT pathway and at the post-translational level through phosphorylation.

## Methods

### Cell culture

K562, KU812, HL60 and Jurkat cell lines, obtained from American Type Culture Collection (ATCC), were cultured in RPMI supplemented with 10% heat-inactivated FBS, 100 units/ml penicillin, 100 μg/ml streptomycin, and 2 mM L-glutamine at 37°C in a 5% CO2 incubator. Approximately 1.5 × 10^5 ^cells were plated in each well of a 6-well plate for drug treatment. BCR-ABL-positive CML patient primary cells (AD155) were obtained with consent and through an approved protocol from the Institutional Review Board (IRB) of the National University of Singapore in accordance with the Helsinki protocol.

### Compounds

Gleevec was a gift from Novartis AG. STAT5 inhibitor (Cat. No. 573108) was obtained from Calbiochem. STAT5 inhibitor was dissolved in dimethyl sulfoxide and diluted to a final concentration of 0.1% dimethyl sulfoxide in all experiments.

### Transfection and infection

Recombinant lentivirus (GFP-hTERT IRES hygro) was produced by co-transfecting 4 μg of pMD.G plasmid, 8 μg of pCMVDR8.91 and 12 μg of lentivector into 293T cells (ATCC) using Lipofectamine 2000 (Invitrogen). Medium containing virus was collected following 24 and 48 h post transfection and filtered through 0.45-μm filters. K562, HL60 and Jurkat cells were incubated with medium containing virus supplemented with 8 μg/ml polybrene for 24 h. Cells were selected with hygromycin for 3-5 days prior to drug treatment.

### Gel-based TRAP assay

Measurement of TA was performed by the PCR-based TRAP (Telomeric repeat amplification protocol) assay, using the TRAPEZE telomerase detection kit (Millipore), according to the manufacturer's instructions. Cells were harvested using ice-cold CHAPS (3-[(3-cholamidopropyl) dimethylammonio]-1-propane sulphonate) lysis buffer and incubated for 30 min on ice. Cells were clarified by centrifugation at 12000 g for 20 min at 4°C. Telomerase extracts were assayed for TA by TRAP analysis. Each reaction was performed in 50 μL reaction mixture containing 10X TRAP reaction buffer, 50X dNTP mix, ^32^P-TS primer, TRAP primer mix and Taq polymerase (5 units/μL). 2-step PCR was performed at 30°C for 30 min and was then subjected to PCR amplification for 27 cycles at 94°C for 30 sec, 59°C for 30 sec each. PCR products were separated by electrophoresis on a 10% urea gel (National Diagnostics) in a T-REX™ Aluminum Backed Sequencer Model S3S (Owl). Gel was transferred using filter papers into a cassette, incubated for 1 week with phosphoimager and was then scanned using Typhoon Trio Imager (Amersham Biosciences). Quantifications were performed using ImageQuant TL (Amersham Biosciences) and TA was normalized with the 36 bp internal PCR control.

### Quantitative telomerase assay

TA was quantified using telomeric repeat amplification protocol (TRAP) as described by the TeloExpress Quantitative Telomerase Detection Kit (XpressBio). Cells were lysed with 50 μl of TeloExpress Lysis buffer and approximately 1 μg of DNA was used for real-time PCR. TA in each sample was calculated based on the comparison with the Ct values of a standard curve generated from 10-fold dilutions of telomerase control (TC) oligo with known copy numbers of the telomeric repeats.

### Telomere length assay

DNA was extracted from the cells using DNeasy Blood & Tissue Kit (Qiagen). Telomere length analysis was carried out using a non-radioactive TeloTAGGG Telomere Length Assay (Roche) as described by the manufacturer. Approximately 1 μg of DNA of each sample was digested with Hinf I/Rsa I enzyme mix and separated by gel electrophoresis. DNA fragments were transferred to nylon membrane (Amersham, GE) by southern transfer and hybridized to digoxigenin (DIG-labeled probe), specific for telomeric repeats. A DIG-specific antibody conjugated to alkaline phosphatase was then used to incubate the membrane and the probe was then visualized by chemiluminescence detection and subsequent exposure to X-ray film (Amsersham, GE). Mean telomeric repeat binding factor (TRF) lengths were determined by comparison to the molecular weight standard provided.

### STELA

XpYp single telomere length assay (STELA) was performed using the methods described by Baird et al. [28]. Total number of telomere bands from the lanes for each sample were pooled and calculated. Telomere shortening was quantified by determining the percentage of telomere bands less than 1.0 kb to the total number of bands in the sample.

### RT-PCR

One step RT-PCR was performed using the Qiagen One Step RT-PCR kit following manufacturer's protocol. The following primers were used: 5'-CGTGGTTTCTGTGTGGTGTC-3' (human TERT forward primer) and 5'-CCTTGTCGCCTGAGGAGTAG-3' (human TERT reverse primer), 5'-GCCTTCCACCGTTCATTCTA-3' (human TER forward primer) and 5'-GCTGACAGAGCCCAACTCTT-3' (human TER reverse primer), 5'-GAGAGACCCTCACTGCTG-3' (GAPDH forward primer) and 5'-GATGGTACATGACAAGGTGC-3' (GAPDH reverse primer). PCR products were run on 2% agarose gel and viewed under UV Gel Doc (BioRad). Quantifications were performed using Quantity One (BioRad).

### Real-time PCR

Reverse transcription was performed using the Promega RT-PCR kit and oligo dT primer as per manufacturer's protocol (Promega). Real-time PCR was performed using Brilliant SYBR Green qPCR Master Mix on the Rotorgene real-time system (Qiagen). The following primers were used for real-time PCR: 5'-GGAGCTGGTGGTTGACTTTC-3' and 5'-CTCCGATTCAGTCCCTTCTG-3' (human BCL2), 5'-ATACCATGATAGCGCCCTTG-3' and 5'-AATCACAGCGAACCTCTGCT-3' (human PI3KCG), 5'-CCCTCGGTGTCCTACTTCAA-3' and 5'-AGGAAGCGGTCCAGGTAGTT-3' (human CCND1), 5'-TGCCAAGAGTCTAGCCCAGT-3' and 5'-TCCACTGTTCATAGGGCACA-3' (human PFKFB4), 5'-ATGCGACAGTTCGTGGCTCA-3' and 5'-ATCCCCTGGCACTGGACGTA-3' (human TERT), 5'-GTGGACCTGACCTGCCGTCT-3' and 5'-GGAGGAGTGGGTGTCGCTGT-3' (human GAPDH). Data were analyzed using the ΔΔCT method.

### Western blotting

Cells were harvested for protein at different time points. Briefly, cells were resuspended in 50 mmol/L Tris-HCl (pH 7.4), 250 mmol/L NaCl, 5 mmol/L EDTA, and 0.1% NP40 containing protease and phosphatase inhibitors. Lysates were cleared by centrifugation at 14,000 rpm for 10 min, and samples were run on SDS-PAGE gels. Western blotting was performed with the following antibodies: rabbit anti-BCR-ABL (Cell Signaling), rabbit anti-hTERT (Epitomics), rabbit anti-pSTAT5 (Tyr694) C11C5 (Cell Signaling), mouse anti-pTyr (Millipore) and phospho-abl (Tyr412) (Millipore). Mouse anti-α-tubulin (Sigma) or Horseradish peroxidase (HRP)-conjugated mouse anti-β-actin (Abcam) or mouse anti-GAPDH (Cell Signaling) were used as loading controls. Immunostaining was detected using ECL Plus Detection Reagent (GE Healthcare).

### Immunoprecipitation

After Gleevec treatment, K562 and HL60 cells were rinsed in cold PBS and lysed in a RIPA buffer. Cell lysate was kept on ice for 10 min and centrifuged for 10 min at 12,000 g, 4°C. 2 μl of anti-hTERT antibody (Abcam) was added to 200 μl of cell lysate and incubated overnight at 4°C. 20 μl of protein A agarose beads (50% bead slurry) was added for 3 h at 4°C. Samples were centrifuged for 30 sec at 4°C. Pellets were washed five times with lysis buffer. Laemmli buffer was added and samples were boiled at 100°C for 5 min. Samples were centrifuged for 1 min at 14,000 g, and supernatants analysed by Western blotting. The tyrosine phosphorylation level of hTERT was examined by anti-phosphotyrosine antibody (Millipore).

### siRNA transfection

siRNA oligos for knockdown of endogenous human STAT5a and STAT5b proteins and Negative Control (Scramble) siRNA were purchased from Ambion. Transfections were performed by Amaxa nucleofection (Lonza), by using program T-016 (K562 cells) or T-019 (HL60 cells) as per manufacturers' instructions.

### Luciferase reporter assay

HeLa cells were seeded in 24-well plates and co-transfected with *hTERT *promoter (-3915 ~ +40) luciferase construct (pGL3- hTERT), pMX-STAT5a, pMX-STAT5b, or pMX (vector control) using Lipofectamine 2000 (Invitrogen). Forty-eight hours following transfection, cells were harvested and subjected to the luciferase assay using the dual-luciferase reporter assay kit (Promega). The pRL-SV40 driving Renilla reniformis luciferase was included in each transfection as a control to normalize the transcriptional activity of hTERT promoter fragments. Standard deviations were derived from three independent experiments.

### Confocal microscopy

K562, HL60 and Jurkat cells were infected with GFP-hTERT IRES hygro expressing virus. Cells were cytospun according to Shandon Cytospin Program and then fixed with 2% paraformaldehyde in PBS, and permeabilized with 0.2% Triton X-100 in PBS. Immunostaining was performed using primary antibodies against fibrillarin (Cell Signaling) followed by appropriate secondary antibody conjugated with Alexa Fluor 568 antibody (Invitrogen). DNA was visualized with 0.2 μg/mL of 4',6-diamidino-2-phenylindole (DAPI) and all images were analyzed using Olympus Fluoview FV100 microscope with 60× objective.

### Illumina microarray analysis

RNA was isolated from K562 and HL60 cells, non-treated or treated with 1 μM of Gleevec for 8 h, using the RNeasy kit with on-column DNase digestion (Qiagen). Quality of RNA from each sample was assessed using the RNAnalyzer (Biorad), all with an RNA integrity number > 7, and 500 ng of RNA was converted to cRNA using the Illumina TotalPrep RNA Amplification kit (Ambion). Labeled cRNA was prepared and hybridized overnight for 18 h to Illumina HumanRef-8_V2 array containing 18,126 human genes, which was then washed and stained with streptavidin-Cy3 (Amersham-Pharmacia Biotech) according to the manufacturer's guidelines (Illumina) and arrays were scanned on a BeadArray Reader (Illumina). Using the Partek software, statistical significance of global gene expression levels were analyzed by Analysis of Variance (ANOVA) at false discovery rate (FDR) < 0.05 and were defined as genes with two-fold up or downregulation. Microarray data is available in MAIME-compliant form at NCBI Gene Expression Omnibus (http://www.ncbi.nlm.nih.gov/geo) under accession number GSE26821.

## Results

### Gleevec specifically inhibits TA in BCR-ABL positive cells

Since telomerase plays a critical role in tumorigenesis, the effects of different drugs on TA are of potential importance [29]. In this study, we found that Gleevec significantly decreased K562 cells viability and proliferation within 48 h (Additional file 1: Figure S1). This result is in agreement with previous studies, which demonstrated that the inhibitory effect of Gleevec on leukemia cells is at least partially due to its inhibitory effect on telomerase activity [23,25,30].

**Figure 1:**
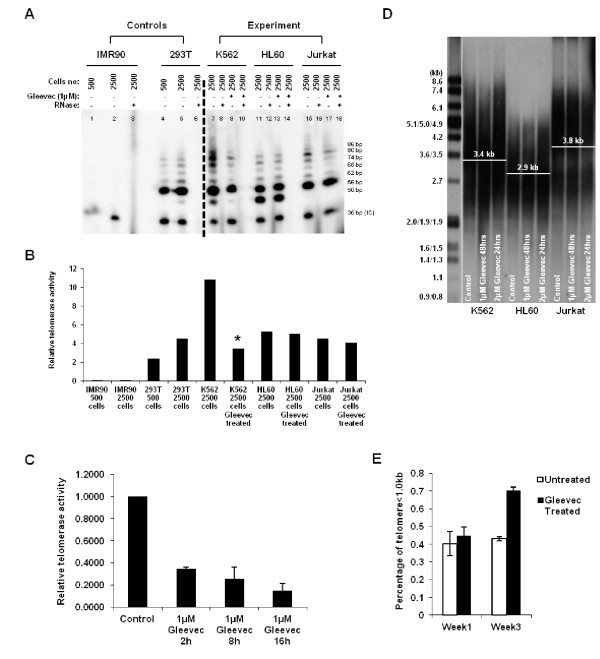
**Gleevec reduces TA in K562 cells**. (**a**) The Gel-based TRAP assay was performed with ^32^P-ATP labeled TS primer using the 2-step PCR conditions: 94°C/30 sec and 59°C/30 sec for 27 cycles. The amount of extract (expressed as cell number) present in telomerase-positive samples (293T, K562, HL60 and Jurkat) and negative controls (IMR90 and samples with RNase) is shown above each lane. K562, HL60 and Jurkat cells were subjected to 1 μM Gleevec treatment for 16 h. (**b**) Normalized relative TA from Gel-based TRAP assay was determined using ImageQuant TL in non-treated and 1 μM Gleevec treated K562, HL60 and Jurkat cells for 16 h. (**c**) Quantitative telomerase assay showing relative TA in K562 cells. Error bars represent standard deviation from three experiments. (**d**) Telomere length assay of K562, HL60 and Jurkat cells under Gleevec treatment. (**e**) Percentage of telomeres less than 1.0 kb as determined by STELA after 1, or 3 weeks with 0.05 μM Gleevec

In order to attest the mechanism of Gleevec on TA and its regulation, TA of BCR-ABL positive (K562) and deficient (HL60 and Jurkat) cells were assessed by gel-based Telomeric Repeat Amplification Protocol (TRAP) assay following 16 h of Gleevec treatment. TRAP results showed that K562 cells have a significantly higher TA (~50% higher) than HL60 or Jurkat cells (Figure 1a and 1b). However, upon 1 μM Gleevec treatment, we observed that TA in K562 cells was reduced by ~70%. Nevertheless, this effect of Gleevec was not evident in BCR-ABL deficient cells, i.e., HL60 and Jurkat cells (Figure 1a and 1b). On the other hand, TRAP results also indicated that Gleevec treatment had no effect on telomerase processivity in both BCR-ABL positive and deficient cells (Figure 1a). To further confirm the effect of Gleevec on TA in K562 cells, Quantitative Telomerase Assay was also performed. Treatment of 1 μM Gleevec for 2 h resulted in a significant reduction of TA in K562 cells, which was consistent with the gel-based TRAP assay result (Figure 1c). To avoid cell specific effects, another BCR-ABL positive cell line, KU812 and BCR-ABL positive CML patient cells, AD155, were used to determine Gleevec effect on TA. Our results also showed a significant decrease in TA in KU812 and AD155 cells under Gleevec treatment (Additional file 1: Figure S2). These results indicate that Gleevec specifically inhibits TA in BCR-ABL positive cells, i.e., K562, KU812, and AD155 cells.

To investigate the effect of Gleevec on telomere length, K562, HL60, and Jurkat cells were treated with Gleevec and telomere lengths were quantified using Southern Blotting. Telomere signals appeared as a broad smear of densities. Mean telomere length of K562, HL60, and Jurkat cells were 3.4 kb, 2.9 kb and 3.8 kb, respectively (Figure 1d) and the result showed that there was no significant change in telomere length upon Gleevec treatment. This could presumably be due to the short treatment period of cells with Gleevec, preferred within 24 h.

We next questioned if Gleevec would induce a longer term effect on telomere length of K562 cells. We used subapoptotic concentrations of Gleevec [31] to treat the cells for a longer period. K562 cells were cultured for up to 3 weeks with 0.05 μM Gleevec. Cells were collected at week 1 and week 3 and then subjected to telomere length determination using the single telomere length assay (STELA) [28]. Telomere shortening was quantified by determining the percentage of telomere bands less than 1.0 kb to the total number of bands in the sample. As shown in Figure 1e, Gleevec did not induce visible telomere length shortening after 1 week treatment. After 3 weeks treatment, however, K562 cells displayed a significantly increased proportion of shortened telomeres (< 1.0 kb), suggesting that Gleevec has a long-term effect on telomere length via inhibiting TA.

### Gleevec specifically downregulates *hTERT *mRNA level in BCR-ABL positive cells

**Figure 2:**
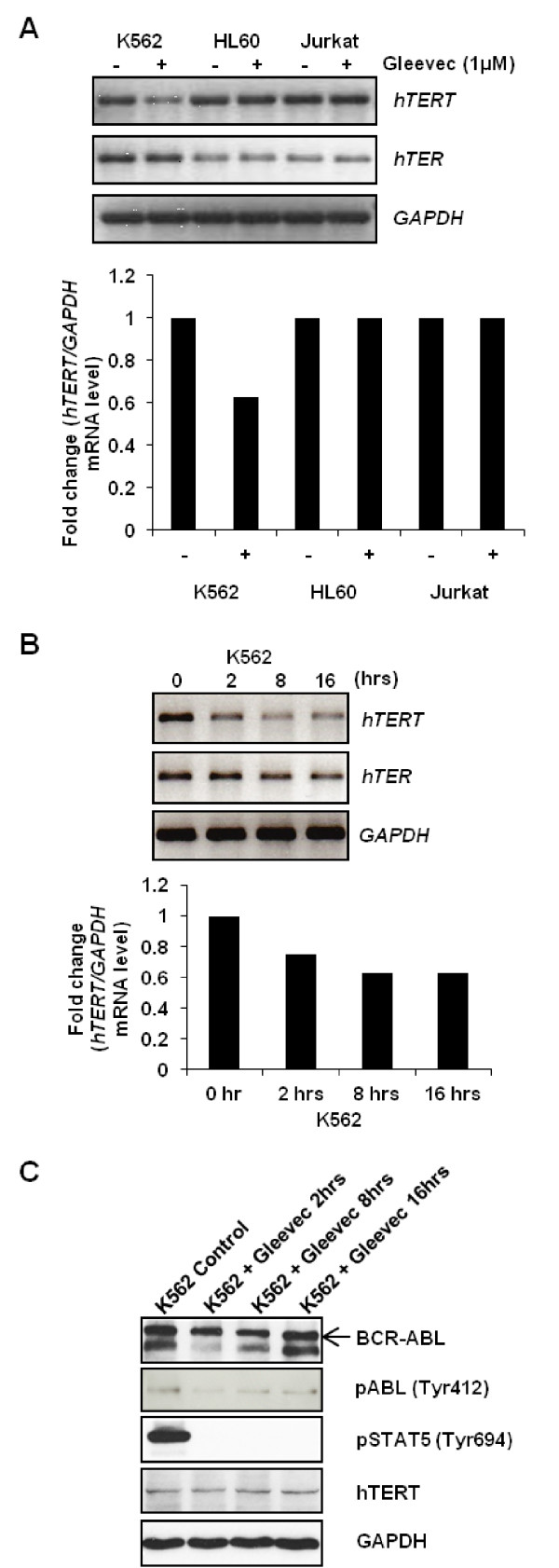
**Gleevec reduces *hTERT *mRNA level in K562 cells**. (**a**) 1 μM Gleevec treatment for 16 h inhibits *hTERT *mRNA level in K562 but not in HL60 and Jurkat cells. Upper Panel: RT-PCR image; Lower Panel: Quantitation of the RT-PCR image. (**b**) *hTERT *mRNA level in K562 cells was gradually reduced with increased incubation time upon 1 μM Gleevec treatment. Upper Panel: RT-PCR image; Lower Panel: Quantitation of the RT-PCR image. (**c**) Endogenous proteins expression level of K562 cells under 1 μM Gleevec treatment for 2, 8 and 16 h

To elucidate the mechanism of Gleevec's inhibitory effect on TA in BCR-ABL positive cells, RT-PCR was performed to quantify *hTERT *and *hTER *mRNA levels in K562, HL60, and Jurkat cells. The basal level of *hTERT *is the same throughout the three cell lines, although the level of *hTER *is higher in BCR-ABL positive K562 cells as compared to BCR-ABL deficient HL60 and Jurkat cells. Gleevec treatment for 16-hour significantly reduced the *hTERT *mRNA level in K562 cells compared to the non-treated control, but the same Gleevec treatment had no effect on *hTERT *mRNA level in BCR-ABL deficient HL60 or Jurkat cells (Figure 2a). Same samples were subjected to real-time PCR for validation of the result shown in RT-PCR. Real-time PCR result also showed reduced *hTERT *mRNA level, by ~60%, upon 16-hour treatment in K562 cells, which showed consistency with the RT-PCR result (Additional file 1: Figure S3). In addition, *hTERT *mRNA level was gradually reduced with increasing Gleevec incubation time, suggesting a direct positive correlation between Gleevec targeted pathway and *hTERT *expression (Figure 2b). However, *hTER *mRNA levels remained unchanged in both the BCR-ABL positive and deficient cells treated with Gleevec (Figure 2a). These data suggested that Gleevec inhibited TA rapidly by specifically reducing *hTERT *mRNA level in BCR-ABL positive K562 cells. Similar result was also shown in KU812 cells by using real-time PCR (Additional file 1: Figure S4).

We next conducted a microarray analysis to examine the gene expression changes between control and 8 h Gleevec-treated cells. We first compared K562 non-treated cells with HL60 non-treated cells and then further analysed the comparison between K562 Gleevec-treated cells with K562 control cells. From our microarray analysis, we noted distinct profiles of gene expression changes in both sets of comparisons. In K562 control cells versus HL60 control cells, a total of 855 and 2182 genes were significantly upregulated and downregulated, respectively (with a *p *value < 0.05 and fold change > or < 2). When comparing K562 Gleevec-treated cells versus K562 control cells, 1114 genes were significantly upregulated and 113 observed to be significantly downregulated (with a *p *value < 0.05 and fold change > or < 2) (Additional file 1: Figure S5). Overlapping genes are listed in Additional file 1 (Table S1). We next sought to identify the gene sets that were enriched in K562 Gleevec-treated group versus K562 control group. This was achieved through the comparison of our array data against known curated gene sets available from Molecular Signatures Database (MSigDB). In agreement to our Western results, our gene set analysis revealed that a number of downregulated genes in K562 Gleevec-treated set were grouped under the JAK/STAT pathway (Table 1A). Three genes were selected from Table 1B and further validated by real-time PCR (Additional file 1: Figure S6-8, Table S2). In particular, we noted a downregulation of *PIK3CG *(also known as *PI3K*), a gene coding for the catalytic component of phosphoinositide-3-kinase (PI3K), a non-receptor tyrosine kinase (Table 1B). A significant difference of almost 3 folds downregulation was observed when K562 cells were treated with Gleevec as compared to K562 control cells (Additional file 1: Figure S7). Though we did not observe any significant downregulation of *hTERT *mRNA in microarray analysis, real-time PCR results showed considerable *hTERT *mRNA downregulation in Gleevec-treated K562 cells (Additional file 1: Figure S9, Table S2). The discordance of *hTERT *gene expression result may be explained by the decreased sensitivity of the microarray gene expression change compared to real-time PCR. But, we noted many upregulated genes that were enriched in gene sets involved in telomere maintenance, telomere extension and telomere ends packaging (Additional file 1: Table S3A & B).

**Table 1:** Comparison between K562 Gleevec-treated group versus K562 control group.

A. K562 Gleevec-treated group versus K562 control group			
**Function**	**Enrichment Score**	**Enrichment *p*-value**	**# genes in list, in group**

KEGG JAK STAT SIGNALING PATHWAY	12.616000	0.000003	8

ST STAT3 PATHWAY	6.219490	0.001990	2

**B. JAK-STAT Signaling Pathway**			

**Gene Symbol**	**Definition**	***p***-**value(K562 Treated vs. K562 Control)**	**Fold**-**Change** **(K562 Treated vs. K562 Control)**

BCL2L1	Homo sapiens BCL2-like 1 (BCL2L1), nuclear gene encoding mitochondrial protein, transcript variant 1, mRNA.	0.000076	-2.336810

CISH	Homo sapiens cytokine inducible SH2-containing protein (CISH), mRNA.	0.000754	-2.926860

CCND1	Homo sapiens cyclin D1 (CCND1), mRNA.	0.000964	-9.480380

SPRED1	Homo sapiens sprouty-related, EVH1 domain containing 1 (SPRED1), mRNA.	0.001425	-3.138930

SOCS1	Homo sapiens suppressor of cytokine signaling 1 (SOCS1), mRNA.	0.003518	-2.455500

IL6	Homo sapiens interleukin 6 (interferon, beta 2) (IL6), mRNA.	0.004541	-2.036640

PIK3CG	Homo sapiens phosphoinositide-3-kinase, catalytic, gamma polypeptide (PIK3CG), mRNA.	0.005740	-2.292180

PIM1	Homo sapiens pim-1 oncogene (PIM1), mRNA.	0.006760	-2.343000

(**a**) Gene set analysis showing enrichment of gene sets under JAK/STAT pathway. These gene sets showed the number of genes that are downregulated in this pathway in K562 Gleevec-treated cells. (**b**) List of downregulated genes related to JAK/STAT pathway in K562 Gleevec-treated cells

Next, we determined whether Gleevec could inhibit hTERT expression at the protein level in K562 cells treated with 1 μM of Gleevec at different time points. Surprisingly, there was no significant alteration in the protein expression level of hTERT upon 16 h of Gleevec treatment (Figure 2c). This could be due to a short half-life of *hTERT *mRNA (~2 h) [32] compared to the long half-life of hTERT protein (~24 h) in K562 cells (Additional file 1: Figure S10) [33], resulting in an indirect correlation between the protein level and the transcriptional level. We have extended the Gleevec treatment beyond 24 h, and we did not observe any marked change in hTERT protein expression level at 24 and 36 h. There is a slight decrease in hTERT protein level at 48 h. This suggested that Gleevec has no significant effect on reducing the rate of hTERT degradation in short-term treatment (Additional file 1: Figure S11). Besides, we observed and confirmed in Figure 2c that Gleevec reduces the tyrosine kinase activity of BCR-ABL by abolishing the phosphorylation of BCR-ABL and therefore eliminates the phosphorylation of STAT5 (Signal Transducer and Activator of Transcription 5). STAT5 is activated by BCR-ABL and is implicated in the pathogenesis of CML. Since STAT5 is a well-known transcriptional activator and has been proven to function as a transcriptional regulator of *hTERT *[34], these evidences led us to hypothesize that BCR-ABL plays a critical role in regulation of hTERT expression through the STAT5 signaling pathway.

### STAT5a plays a critical role in *hTERT *gene expression in K562 cells

**Figure 3:**
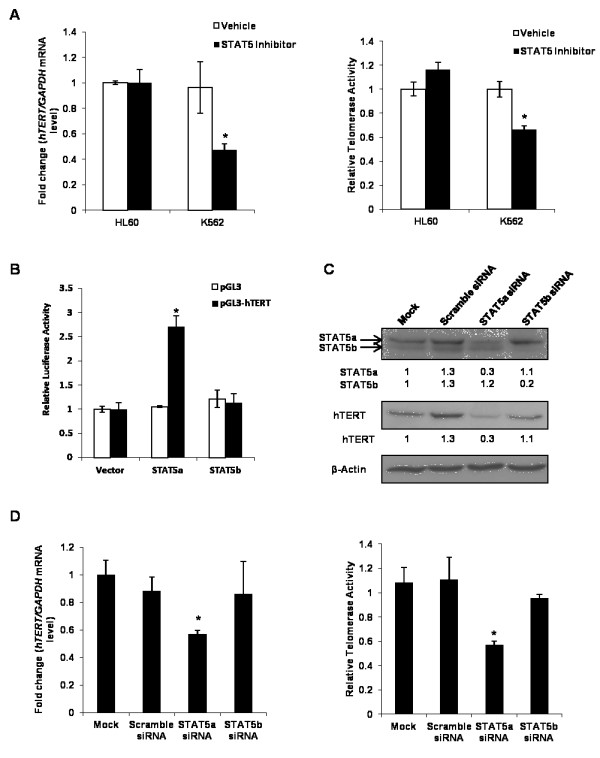
**STAT5a plays important role in *hTERT *mRNA expression**. (**a**) HL60 and K562 cells were treated with vehicle (DMSO) or STAT5 inhibitor (50 μM) for 48 h, after that cells were harvested. Expression of *hTERT *was measured using real-time PCR and normalized to the expression of GAPDH. TA was determined by quantitative telomerase assay. (**b**) K562 cells were co-transfected with empty vector (pGL3) or with a luciferase construct containing the *hTERT *promoter (pGL3-hTERT) together with empty expression vector (pMX) or STAT5a or STAT5b expression construct (pMX-STAT5a or pMX-STAT5b). Luciferase activity was determined 48 h after transfection and was normalized to empty expression vector. Each luciferase assay was repeated 3 times. (**c**) K562 cells were transfected with Scramble siRNA (non-targeting siRNA), STAT5a siRNA, STAT5b siRNA, or non-transfected (Mock). Cells were lysed after 72 h post-transfection. Total amount of STAT5a, STAT5b and hTERT were shown by Western blotting, and quantitated by normalization over the expression of β-Actin using ImageJ. (**d**) After 72 h post-transfection, K562 cells were collected and *hTERT *mRNA expression level and TA were measured by real-time PCR and quantitative telomerase assay, respectively

STAT5 inhibitor was used to examine the specific role of STAT5 in the expression of *hTERT *mRNA in BCR-ABL positive or negative cells. K562 and HL60 cells were treated with either STAT5 inhibitor or vehicle. After 48 h, cells were subjected to determination of *hTERT *mRNA and TA. Real-time PCR revealed that STAT5 inhibitor treatment caused a significant decrease in expression of hTERT in K562 cells, but not in HL60 cells. Moreover, we found that STAT5 inhibitor specifically inhibited TA in K562 cells (Figure 3a).

In order to investigate whether STAT5 could activate the *hTERT *gene promoter, we examined *hTERT *promoter activation by STAT5 using a luciferase reporter assay. A 3.9 kb fragment of the human wild-type *hTERT *promoter was fused to the pGL3-basic luciferase reporter vector [35]. HeLa cells were transiently co-transfected with the *hTERT *full-length promoter construct (pGL3-hTERT) and pMX-STAT5a or pMX-STAT5b, while empty vector was used as a control [36]. Activation of the *hTERT *promoter was measured by luciferase activity. HeLa cells transfected with STAT5a showed a 2.7-fold increase in luciferase activity compared to control cells, while the induction of luciferase activity in the presence of exogenous STAT5b was not statistically significant (Figure 3b). This indicated that the *hTERT *promoter was significantly activated by STAT5a, but not STAT5b.

Next, we verified the importance of STAT5 in *hTERT *gene expression by siRNA assay. K562 cells were transfected with STAT5a siRNA, STAT5b siRNA or scramble siRNA, respectively. The ability and specificity of STAT5a and STAT5b siRNAs were first examined by immunoblotting. Figure 3c showed the scramble siRNA did not affect the expression of STAT5a or STAT5b. As shown in Figure 3c, when STAT5a protein level was 70% reduced, *hTERT *mRNA levels, together with TA, were clearly downregulated after 72 h of post-transfection with STAT5a siRNA, whereas the STAT5b siRNA, which reduced STAT5b protein level significantly by 80%, did not affect *hTERT *mRNA levels as well as TA (Figure 3d). In agreement with these results, we also found knockdown of STAT5a, but not STAT5b, resulted in marked reduction in hTERT protein level (Figure 3c). When same experiments were carried out in HL60, BCR-ABL negative cells, STAT5a silencing showed no effect on *hTERT *mRNA expression and TA (Additional file 1: Figure S12).

Taken together, these data strongly suggested that activated STAT5a, but not STAT5b, plays a critical role in telomerase regulation in K562, BCR-ABL positive cells. These findings also indicated that BCR-ABL could regulate TA by transcriptional control of *hTERT *mRNA level through the JAK-STAT pathway.

### Gleevec regulates human TA and hTERT phosphorylation through inhibition of BCR-ABL kinase activity

**Figure 4:**
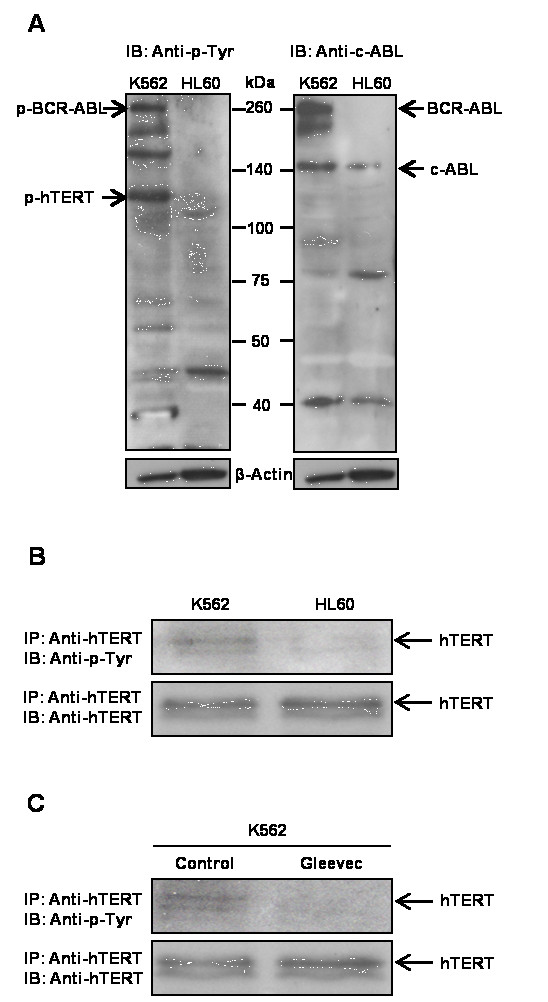
**Post-translational regulation and phosphorylation of BCR-ABL on hTERT**. (**a**) Global protein tyrosine-phosphorylation level and BCR-ABL expression in K562 and HL60 cells. (**b**) Phosphorylation of hTERT at tyrosine residues was higher in K562 cells compared to HL60 cells. (**c**) Gleevec treatment led to a loss of phosphorylation of hTERT at tyrosine residues

Some studies showed that protein kinase C (PKC) α and AKT/protein kinase B (PKB) can upregulate human TA through phosphorylation of hTERT [20,21]. Given that BCR-ABL functions as a tyrosine kinase, we questioned whether BCR-ABL could also directly enhance TA through phosphorylation of hTERT. To address this question, we analyzed the phosphorylation level of hTERT using anti-phosphotyrosine antibody in both BCR-ABL positive (K562) and BCR-ABL deficient (HL60) cells. Compared to HL60 cells, the tyrosine phosphorylation level in K562 cells was markedly increased (Figure 4a), suggesting that the increase in tyrosine phosphorylation is due to BCR-ABL tyrosine kinase activity, which was confirmed by the expression of BCR-ABL shown only in K562 cells. Interestingly, we found a significant increase in tyrosine-phosphorylation at the corresponding molecular weight of hTERT (122 kDa) in K562 cells compared to HL60 cells. This result led us to consider that hTERT could be phosphorylated at tyrosine residues by BCR-ABL in K562 cells. To evaluate this possibility, hTERT was immunoprecipitated by anti-hTERT antibody from both K562 and HL60 cell lysates and resolved by SDS-PAGE followed by immunoblotting with anti-phosphorylation antibody. We found that hTERT tyrosine phosphorylation was significantly elevated in K562 cells compared to HL60 cells (Figure 4b). As the expression level of hTERT was similar in both cells, the result suggested that hTERT could be presumably phosphorylated by BCR-ABL.

To further determine whether BCR-ABL phosphorylates hTERT, we treated K562 cells with 1 μM Gleevec, and evaluated the phosphorylation status of hTERT. If hTERT is a substrate of BCR-ABL, we would expect Gleevec treatment to decrease the phosphorylation level of hTERT and its activity. As shown in Figure 4c, Gleevec treatment resulted in almost complete inhibition of hTERT phosphorylation at tyrosine residues compared to control cells. To demonstrate that the decrease in tyrosine phosphorylation of hTERT was not due to reduced hTERT expression level, western blot was performed and we did not observe a difference in hTERT expression level in Gleevec-treated K562 cells compared to control cells (Figure 4c). We also examined the interaction between BCR-ABL and hTERT using immunoprecipitation assay. Surprisingly, there was no evidence of direct interaction between BCR-ABL and hTERT (data not shown).Overall, these results suggested that BCR-ABL can regulate TA by post-translational modification of hTERT through tyrosine phosphorylation.

### Gleevec inhibits hTERT nucleoli translocation in K562 BCR-ABL positive cells

**Figure 5:**
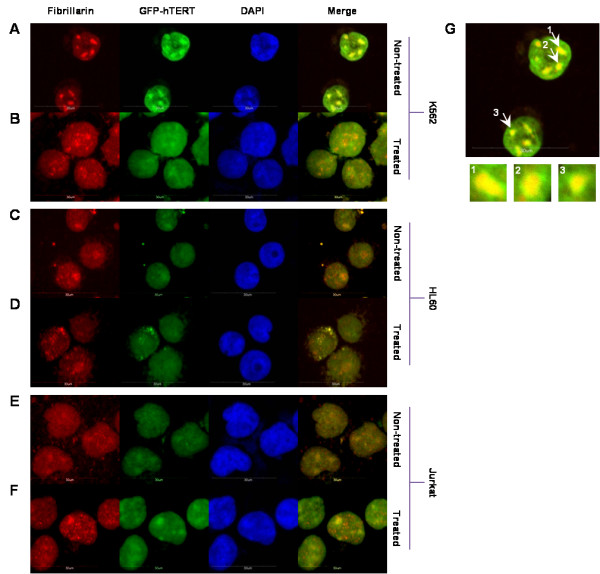
**Gleevec treatment of K562 (BCR-ABL positive) cells induces translocation of telomerase out of the nucleoli**. K562, HL60 and Jurkat cells were infected with GFP-hTERT. Representative immunofluorescence images of (**a**) K562 GFP-hTERT non-treated cells; (**b**) K562 GFP-hTERT treated cells with 1 μM Gleevec for 16 h; (**c**) HL60 GFP-hTERT non-treated cells; (**d**) HL60 GFP-hTERT treated cells with 1 μM Gleevec for 16 h; (**e**) Jurkat GFP-hTERT non-treated cells; and (**f**) Jurkat GFP-hTERT treated cells with 1 μM Gleevec for 16 h. (**g**) Representative enlarged image of GFP-hTERT infected K562 cells displaying colocalization of GFP-hTERT with fibrillarin. Blue: DAPI; Green: GFP-hTERT; Red: Fibrillarin. All images were taken using Olympus Fluoview FV1000. Bar, 30 μm

It is known that phosphorylation of hTERT is important for its nuclear translocation [37]. We subsequently examined the localization of hTERT in K562, HL60, and Jurkat with and without Gleevec treatment. Confocal microscopy was carried out to study Gleevec's effect on hTERT cellular distribution in K562, HL60, and Jurkat cells. These three cell lines were infected with GFP-hTERT as the endogenous level of hTERT could not easily be detected in these cells by immunofluoresence microscopy. They were then either left untreated (Figure 5a, c and 5e) or treated with Gleevec for 16 h (Figure 5b, d and 5f). Images were merged for two colors: GFP-hTERT (green) and fibrillarin (red), the latter was used as a marker for nucleoli while the nucleus was stained with DAPI. A concentrated localization of hTERT was observed in nucleoli of non-treated K562 cells (Figure 5a and 5g), but not in HL60 and Jurkat cells (Figure 5c and 5e). Gleevec treatment induced dissociation of hTERT from nucleoli of K562 cells to nucleoplasm (Figure 5b). This finding indicated that hTERT could partly have translocated into the nucleoplasm or was prevented from binding to nucleoli upon Gleevec treatment. In contrast, HL60 and Jurkat cells showed that hTERT was mainly dispersed in the nucleoplasm but not concentrated in nucleoli (Figure 5c-f).

## Discussion

Previous studies have suggested that treatment of Gleevec could inhibit TA through suppressing *hTERT *mRNA level and hTERT phosphorylation level; the latter is regulated by serine/threonine protein kinase AKT [20,21]. However, the mechanism by which Gleevec inhibits TA in BCR-ABL positive cells remains largely unknown. Given the clinical significance of BCR-ABL in leukemia treatment, we sought to investigate the roles of BCR-ABL in CML and its relationship with telomerase regulation, in order to facilitate in the development of better anti-CML drugs. We found that Gleevec inhibits TA through BCR-ABL by two separate mechanisms: (1) by reducing the *hTERT *mRNA level via suppressing BCR-ABL mediated STAT5 signaling pathway; (2) by inhibiting phosphorylation of hTERT that can reduce TA and induce hTERT cellular translocation.

Our RT-PCR results showed that *hTERT *mRNA is dramatically reduced in the presence of Gleevec. The reduction of expression is only found in *hTERT *but not in *hTER *of telomerase in K562 cells. This implies that Gleevec only affects the catalytic component of telomerase. Moreover, Gleevec treatment of K562 cells resulted in a significant decrease in TA but has no effect on the processivity of the telomerase. Our results are consistent with previous findings that TA is inhibited in BCR-ABL-positive cells by Gleevec and this inhibition is specific to telomerase [25].

It is known that telomerase inhibition can reduce telomere length to a critical threshold resulting in senescence and/or apoptosis. We examined Gleevec's effect on telomere length in K562 cells and observed telomere shortening following 3 weeks of exposure to sub-apoptotic concentrations of Gleevec, while short-term Gleevec treatment showed no significant effect on telomere length. The efficacy of long-term telomerase inhibition suggests that Gleevec may inhibit K562 cell growth and proliferation by modulating telomere length.

From the microarray analysis, we found that *PI3K *was downregulated in the JAK/STAT signaling pathway in Gleevec-treated K562 cells as compared to the K562 control group. Previous study has shown that BCR-ABL activates PI3Ks and extracellular signals to produce phosphatidylinositol-3,4,5-trisphosphate (PIP_3_), which is a second messenger that activates and recruits downstream effector proteins such as the serine/threonine kinase AKT [38]. Thus, this suggests that the downregulation of PI3K is due to the inhibition of BCR-ABL tyrosine kinase activity via the JAK/STAT pathway upon Gleevec treatment in K562 cells, which may ultimately reduce TA in these cells.

STAT family proteins function as downstream effectors of a variety of cytokines and growth factors. STAT factors transmit signals to the nucleus where they bind to specific DNA promoter sequences and thereby regulate gene expression [39]. Numerous studies have demonstrated that constitutively activated STAT factors, particularly STAT3 and STAT5, have been found in a wide variety of human tumors, including blood malignancies (leukemias, lymphomas) [40,41]. Constitutively activated STAT factors are linked to persistent activity of tyrosine kinases, such as BCR-ABL, Src, and many others.

In this study, we observed and confirmed that STAT5 was phosphorylated only in BCR-ABL positive K562 cells and 2-hour Gleevec treatment completely abolished the phosphorylation of STAT5, which is in agreement with previous findings describing that STAT5 pathway is constitutively activated by p210 BCR-ABL and p190 BCR-ABL in leukemic cells [42,43]. BCR-ABL directly phosphorylates STAT5 at tyrosine residues and promotes dimerization of phosphorylated STAT5 followed by nuclear translocation of the dimers that then promote activation of downstream target genes, which are important to induce or maintain cancer cell growth and survival. A previous report showed that inhibition of BCR-ABL, as well as STAT5, by a selective inhibitor, suppressed cell proliferation and induced apoptosis in the BCR-ABL/STAT5 double positive K562 CML cell line, while this inhibitor had no effect on either a BCR-ABL-negative/STAT5-positive or a BCR-ABL/STAT5 double-negative myeloid cell line, suggesting that the STAT5 signaling pathway leading to growth and survival is BCR-ABL-dependent [44]. Some studies demonstrated that STAT5 activation is absolutely essential for leukemic cells because STAT5 activation leads to increased expression of genes driving cell cycle progression and promoting survival [45,46], but it still remains unclear whether STAT5 is involved in regulating telomerase, which plays critical role in tumor cell growth and proliferation.

We present here several lines of evidence for a role of STAT5 in telomerase regulation in BCR-ABL positive K562 cells. We have shown that Gleevec treatment reduced STAT5 phosphorylation, which coincides with a decrease in *hTERT *mRNA expression. We also found that STAT5 inhibitor selectively suppressed *hTERT *mRNA expression and TA in BCR-ABL positive K562 cells. It has been known that STAT5 comprises of two highly homologous genes encoding STAT5a and STAT5b [47]. Although these two STAT proteins share considerable functional overlap, gene-disruption experiments have revealed that STAT5a and STAT5b are functionally not redundant [48,49]. Previous studies demonstrated that STAT5a mediates prolactin signaling along with mammary gland development [48,50], whereas knockdown of STAT5b abrogates sexually dimorphic liver gene regulation and is associated with loss of male characteristic body growth rates [51]. In this study, we demonstrated that STAT5a, but not STAT5b, expression and phosphorylation correlated with *hTERT *gene expression and TA. More importantly, knockdown of STAT5a as well as Gleevec treatment severely reduced *hTERT *gene expression and TA in BCR-ABL positive K562 cells but not in BCR-ABL negative HL60 cells. These results strongly support the notion that constitutive activation of STAT5a is likely to make a significant contribution to the telomerase regulation in BCR-ABL positive CML cells, suggesting that STAT5a could be an attractive target for the treatment of CML, especially in cases of multiple inhibitor-resistant CML. Our findings are in accordance with recent reports which demonstrated that STAT5 accounts for the resistance against Gleevec and inhibition of STAT5 can effectively decrease survival of CML cells resistant to tyrosine kinase inhibitors [52,53].

It is known that protein phosphorylation is an important post-translational regulation controlling protein structure and function [54]. Some reports indicated that PKC can stimulate TA through phosphorylation of hTERT, while TA was markedly inhibited in the presence of protein phosphatase 2A (PP2A) [55]. These findings suggest that PKC and PP2A are involved in reciprocally controlling TA through phosphorylation and dephosphorylation. In addition to PKC, AKT was also found to phosphorylate the serine residue at position 824 of hTERT and stimulate TA [20]. In our study, immunoprecipitation assay demonstrated that the hTERT tyrosine phosphorylation level was higher in K562 cells compared to HL60 cells and Gleevec treatment could effectively abrogate hTERT tyrosine phosphorylation in K562 cells as well as TA inhibition, suggesting that BCR-ABL could also phosphorylate hTERT and this phosphorylation may be important for TA maintenance and regulation. However, further investigations are necessary to determine which tyrosine could be the substrate of BCR-ABL. Previous results demonstrated that c-ABL, a non-receptor tyrosine kinase, can directly interact with hTERT and inhibit TA following phosphorylation of hTERT [56]. This suggests that c-ABL plays a negative role in regulating telomerase function and as such we determined whether c-ABL could affect TA and hTERT protein level in c-ABL^-/- ^mouse embryonic fibroblasts (MEFs). We found that there was no significant effect on TA and hTERT expression by the c-ABL deficiency (Additional file 1: Figure S13).

Previously, Liu and colleagues reported that phosphorylation of hTERT may be an important mechanism to regulate hTERT subcellular translocation from the cytosol to the nucleus [37]. Presumably, the translocation of hTERT from a non-functional cytosolic location to a physiologically relevant nuclear location may play an important role in regulating TA in cells. As we revealed here that hTERT could be phosphorylated by BCR-ABL, we next questioned whether BCR-ABL could also govern hTERT translocation in different cellular compartments. Our confocal images have shown that hTERT in K562 BCR-ABL positive cells were localized and concentrated in nucleoli at normal conditions. Under Gleevec treatment, most of the hTERT dissociated from nucleoli into the nucleoplasm. In contrast, this phenomenon was not observed in HL60 and Jurkat, BCR-ABL deficient cells. This implies that Gleevec treatment could possibly inhibit phosphorylation of hTERT, induce hTERT translocation and thereby decrease telomerase enzyme assembly and subsequent activity.

We suppose that hTERT could be phosphorylated by BCR-ABL directly since hTERT tyrosine phosphorylation level was found elevated in K562 cells by immunoprecipitation assay (Figure 4b). In addition, the expression level of hTERT was similar in both cells. This result suggests that hTERT could be phosphorylated by BCR-ABL. Moreover, as shown in Figure 4c, Gleevec treatment resulted in near-elimination of hTERT phosphorylation at tyrosine residues compared to control. We also demonstrated that the decrease in tyrosine phosphorylation of hTERT was not due to reduced hTERT expression level (Figure 4c). However, our immunoprecipitation results showed that neither c-ABL nor BCR-ABL interacts with hTERT directly, which contradicts to a previous study that reported the association of c-ABL with hTERT [56]. This may due to the low affinity binding of BCR-ABL to hTERT or their transient interaction. A previous study has shown that BCR-ABL is a large protein mainly found in the cytoplasm, whereas hTERT is mostly localized in the nucleus, excluding the possibility of a direct association between the two proteins [25]. This also implies that there may be another indirect regulation of BCR-ABL on hTERT, which may require alternative pathways or through some other intermediate proteins.

**Figure 6:**
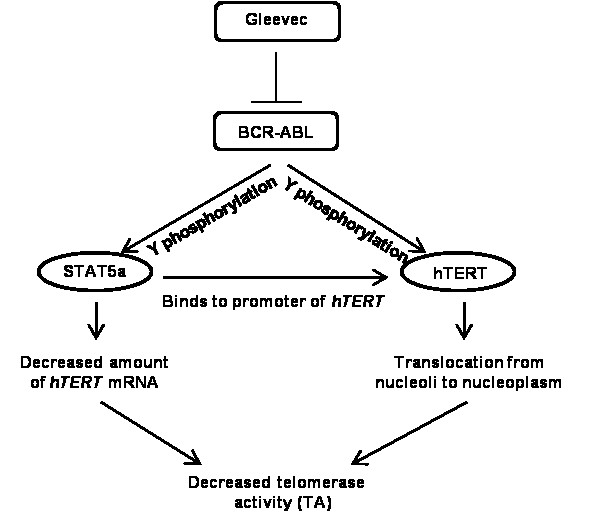
**Hypothetical model of BCR-ABL tyrosine kinase regulation on TA in K562 cells**.

Taken together, we show here that BCR-ABL inhibition by Gleevec treatment has a significant impact on telomerase regulation based on our findings (Figure 6). Our study reveals a link between transcription factor STAT5a and *hTERT *gene expression in BCR-ABL positive CML cell lines. Inhibition of BCR-ABL, and thus STAT5a, by Gleevec leads to reduced TA and *hTERT *mRNA expression as well as downregulation of hTERT phosphorylation at tyrosine residues at the post-translational level. In addition to that, we also found that BCR-ABL might regulate TA through the Wnt signaling pathway (unpublished data). These findings support the notion that telomerase expression and activity could be regulated at multiple levels by the same protein. Shuttling of telomerase in and out of nucleoli induced by Gleevec treatment provides a new insight on BCR-ABL regulated TA.

The introduction of Gleevec has revolutionized the treatment of CML. Despite significant hematologic and cytogenetic responses, there has been concern over the emergence of resistance to Gleevec, which is mostly due to point mutations in the BCR-ABL kinase domain. One such mutation, T315I, renders CML cells completely resistant not only to Gleevec but also to second generation BCR-ABL inhibitors nilotinib and dasatinib [57]. This has spurred the interest in developing novel tyrosine kinase inhibitors or treatment strategies to overcome the mechanisms of resistance that have led to treatment failures. Our findings showed that STAT5, more particularly, STAT5a, plays a critical role in TA regulation, suggesting that inhibition of STAT5a in combination with BCR-ABL may provide an alternative approach for treatment of leukaemia, especially in patients who are resistant to tyrosine inhibitors. Knockdown and inhibition of constitutively active STAT5 has been implicated in growth suppression in CML cells but not in normal cells [58,59]. Such a combination may allow less dose of each drug, and therefore decrease side effects. More importantly, this strategy can decrease the emergence of drug-resistant cells.

## Conclusions

Our data suggest that BCR-ABL can regulate TA at multiple levels, including transcription, post-translational level, and proper localization. Therefore, suppression of cell growth and induction of apoptosis by Gleevec treatment may be partially due to TA inhibition. More importantly, we have identified STAT5a as critical mediator of the *hTERT *gene expression in BCR-ABL positive CML cells, suggesting that targeting STAT5a may be a promising therapeutic strategy for BCR-ABL positive CML patients, especially for resistant cases due to mutants of BCR-ABL.

## Abbreviations

TA: telomerase activity; CML: chronic myeloid leukemia; ALL: acute lymphoblastic; leukemia; BCR: breakpoint cluster region; TRAP: telomeric repeat amplification protocol; STELA: single telomere length assay; Ph: Philadelphia chromosome

## Competing interests

The authors declare that they have no competing interests.

## Authors' contributions

JHC, YZ and WHT carried out the experiments. JHC, YZ and XW participated in the design of the study, its coordination, and helped to draft the manuscript. In addition, technical guidance from WJC, BL and XW lead to successful experiments. All authors approved the final manuscript.

## Pre-publication history

The pre-publication history for this paper can be accessed here:

http://www.biomedcentral.com/1471-2407/11/512/prepub

## Supplementary Material

Additional file 1 **Supplemental data**. Regulation of hTERT by BCR-ABL at multiple levels in K562 cells. Click here for file
