# Supplementary material for: Regulation of hTERT by BCR-ABL at multiple levels in K562 cells
Source: BMC Cancer. 2011 Dec 9;11:512. doi: 10.1186/1471-2407-11-512 (PMC3259104; doi:10.1186/1471-2407-11-512)
Supplement: Additional file 1 — Supplemental data. Regulation of hTERT by BCR-ABL at multiple levels in K562 cells. [file 1471-2407-11-512-S1.PDF]

Supplemental Data

REGULATION OF hTERT BY BCR-ABL AT MULTIPLE LEVELS IN K562 CELLS
Juin Hsien Chai, Yong Zhang, Wei Han Tan, Wee Joo Chng, Baojie Li, and Xueying Wang

Supporting figures and tables

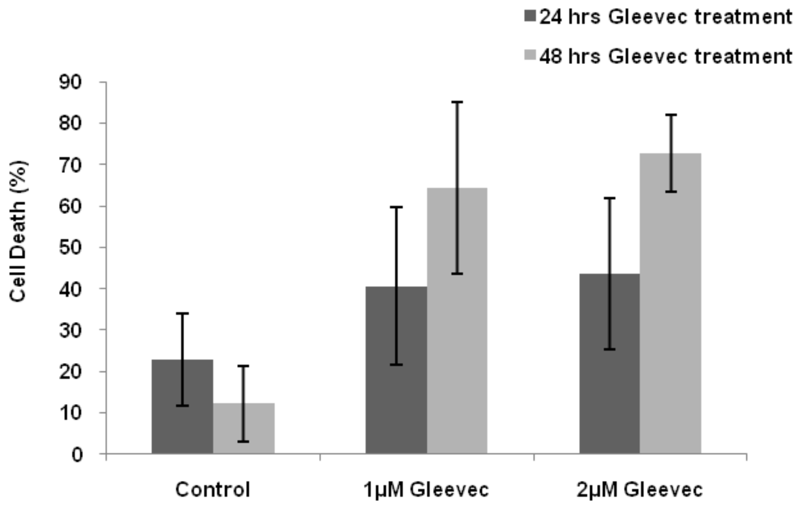

Fig. S1. Percentage of cell death of K562 cells measured by trypan blue staining under Gleevec treatment for 24 and 48 hours. Cells count using hemocytometer. Dead cells are stained blue under trypan blue and are counted as a percentage of total cells. Error bars represent standard deviation from three independent experiments.

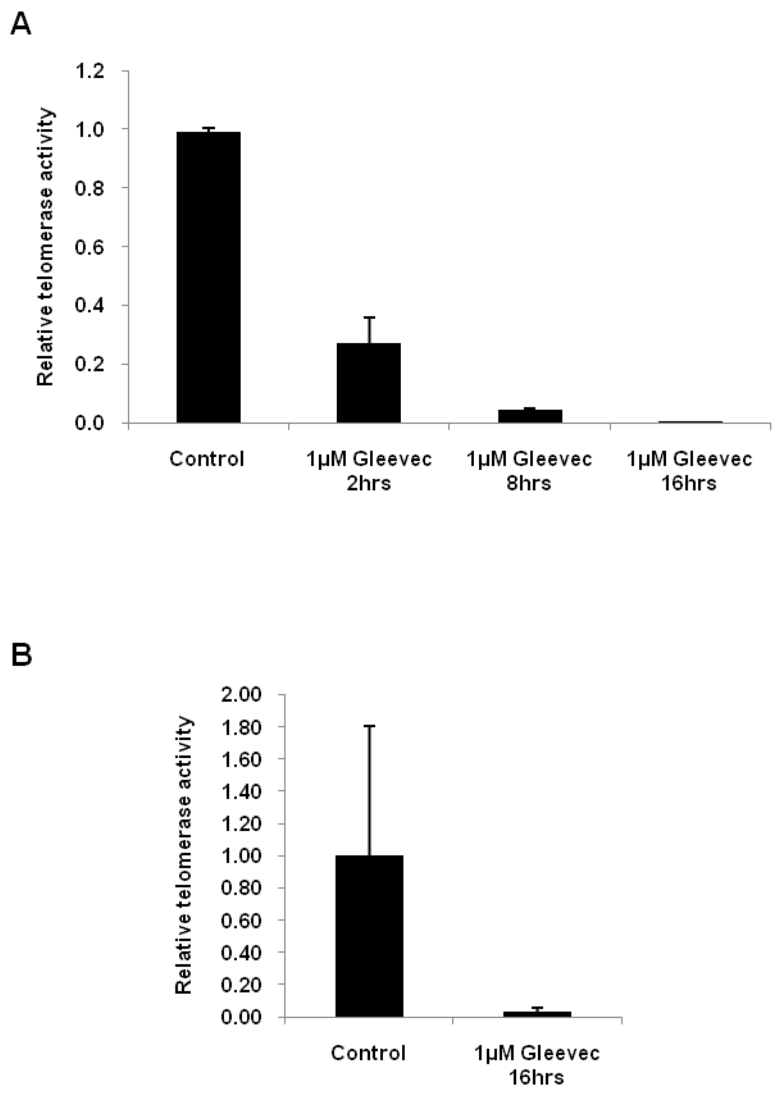

Fig. S2. Gleevec specifically inhibits TA in BCR-ABL positive cells. *A.* Quantitative telomerase assay showing relative TA in KU812 cells. *B.* Quantitative telomerase assay showing relative TA in BCR-ABL positive CML patient cells, AD155. Error bars represent standard deviation from three independent experiments.

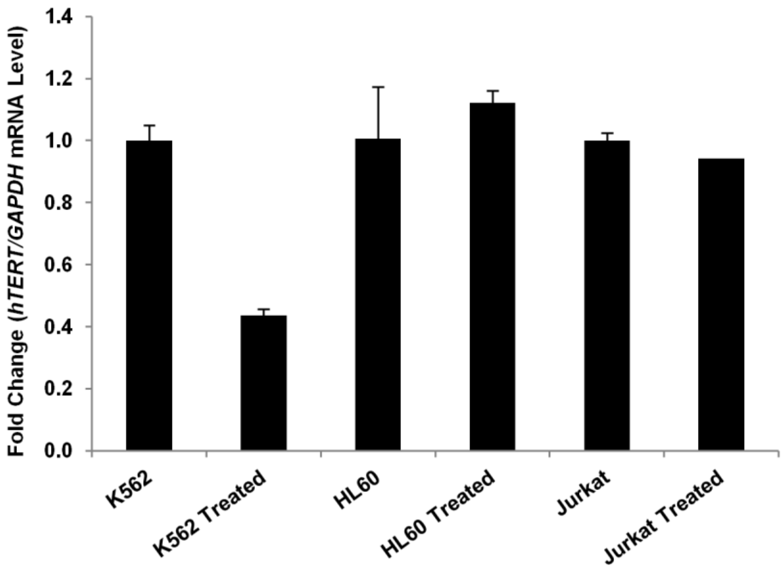

<u>Fig. S3.</u> Fold change of *hTERT* mRNA level following quantitative real-time PCR analysis in K562, HL60 and Jurkat cells under 1μM Gleevec for 16 hours. Error bars represent standard deviation from two replicates.

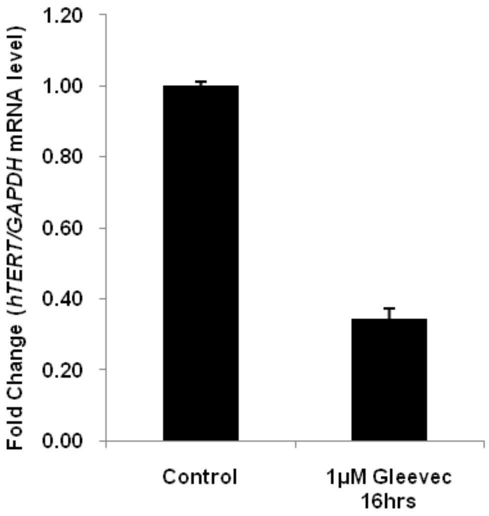

<u>Fig. S4.</u> Fold change of *hTERT* mRNA level following quantitative real-time PCR analysis in KU812 cells under 1μM Gleevec for 16 hours. Error bars represent standard deviation from three independent experiments.

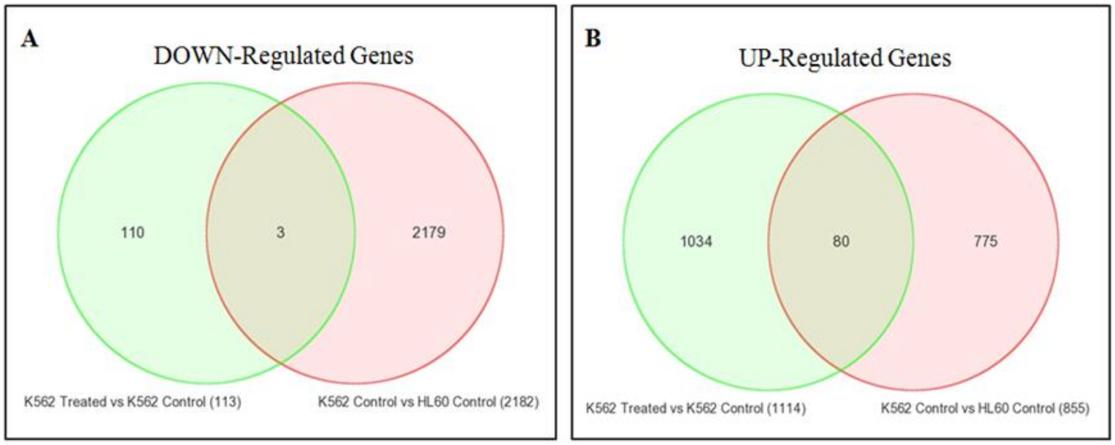

Fig. S5. Comparison of deregulated genes in K562 Treated vs. K562 Control and K562 Control vs. HL60 Control. *A.* Number of downregulated genes observed in K562 Treated vs. K562 Control and K562 Control vs. HL60 Control. *B.* Number of upregulated genes observed in K562 Treated vs. K562 Control and K562 Control vs. HL60 Control.

A Overlapping downregulated genes (K562 Treated vs. K562 Control and K562 Control vs. HL60 Control).

Gene Symbol	Definition	p-value (K562 Control vs. HL60 Control)	Fold-Change (K562 Control vs. HL60 Control)	p-value (K562 Treated vs. K562 Control)	Fold-Change (K562 Treated vs. K562 Control)
CARD9	Homo sapiens caspase recruitment domain family, member 9 (CARD9), mRNA.	0.00072	-3.96086	0.00260	-2.01502
CCND1	Homo sapiens cyclin D1 (CCND1), mRNA.	0.00006	-3.21673	0.00096	-9.48038
AKNA	Homo sapiens AT-hook transcription factor (AKNA), mRNA.	0.00084	-3.40182	0.00032	-2.07818

B Top 10 overlapping upregulated genes (K562 Treated vs. K562 Control and K562 Control vs. HL60 Control).

Gene Symbol	Definition	p-value (K562 Control vs. HL60 Control)	Fold-Change (K562 Control vs. HL60 Control)	p-value (K562 Treated vs. K562 Control)	Fold-Change (K562 Treated vs. K562 Control)
MLLT10	Homo sapiens myeloid/lymphoid or mixed-lineage leukemia (trithorax homolog, Drosophila); translocated to, 10 (MLLT10), transcript variant 1, mRNA.	0.00246	2.32459	0.00366	2.85141
MOCOS	Homo sapiens molybdenum cofactor sulfurase (MOCOS), mRNA.	0.00120	3.33959	0.00512	2.00079
ABHD5	Homo sapiens abhydrolase domain containing 5 (ABHD5), mRNA.	0.00348	2.04871	0.00334	3.02675
OAT	Homo sapiens ornithine aminotransferase (gyrate atrophy) (OAT), nuclear gene encoding mitochondrial protein, mRNA.	0.00050	6.02091	0.00172	5.17149
ATP8B2	Homo sapiens ATPase, Class I, type 8B, member 2 (ATP8B2), transcript variant 1, mRNA.	0.00205	2.22863	0.00173	2.11048
OR51B4	Homo sapiens olfactory receptor, family 51, subfamily B, member 4 (OR51B4), mRNA.	0.00031	6.44212	0.00611	2.01144
P4HA2	Homo sapiens procollagen-proline, 2-oxoglutarate 4-dioxygenase (proline 4-hydroxylase), alpha polypeptide II (P4HA2), transcript variant 2, mRNA.	0.00269	3.53530	0.00284	2.69403
PAQR8	Homo sapiens progestin and adipoQ receptor family member VIII (PAQR8), mRNA.	0.00460	3.18762	0.00637	2.10702
B3GNT1	Homo sapiens UDP-GlcNAc:betaGal beta-1,3-N-acetylglucosaminyltransferase 1 (B3GNT1), mRNA.	0.00505	2.08831	0.00703	2.03021
PFKFB4	Homo sapiens 6-phosphofructo-2-kinase/fructose-2,6-biphosphatase 4 (PFKFB4), mRNA.	0.00020	5.82182	0.00066	3.02128

Table S1. Overlapping deregulated genes in the comparison between K562 Treated vs K562 Control set and K562 Control vs HL60 Control set. *A.* Overlapping downregulated genes (K562 Treated vs K562 Control and K562 Control vs HL60 Control). *B.* Top 10 overlapping upregulated genes (K562 Treated vs K562 Control and K562 Control vs HL60 Control).

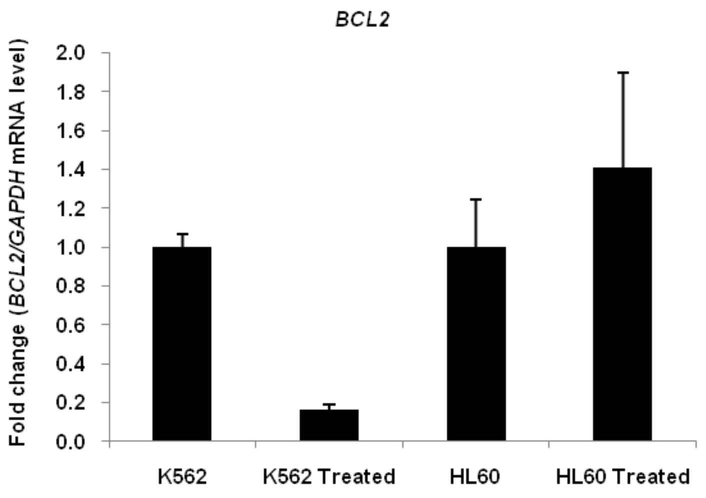

Fig. S6. Fold change of *BCL2* mRNA level following quantitative real-time PCR analysis in K562 and HL60 cells. Error bars represent standard deviation from three independent experiments.

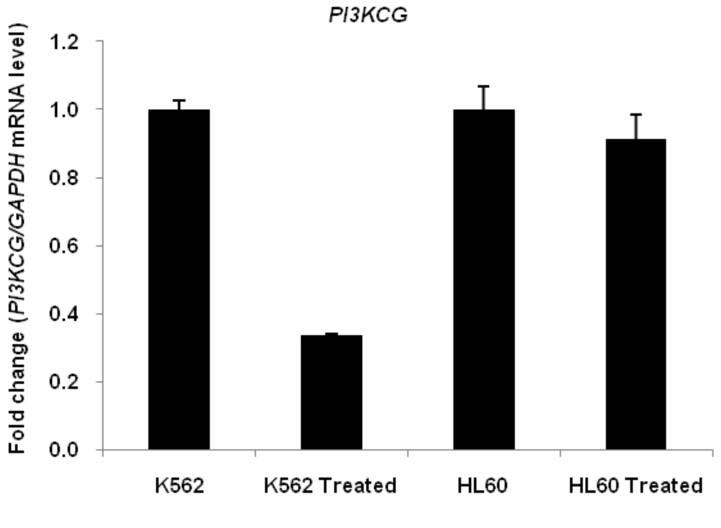

Fig. S7. Fold change of *PI3KCG* mRNA level following quantitative real-time PCR analysis in K562 and HL60 cells. Error bars represent standard deviation from three independent experiments.

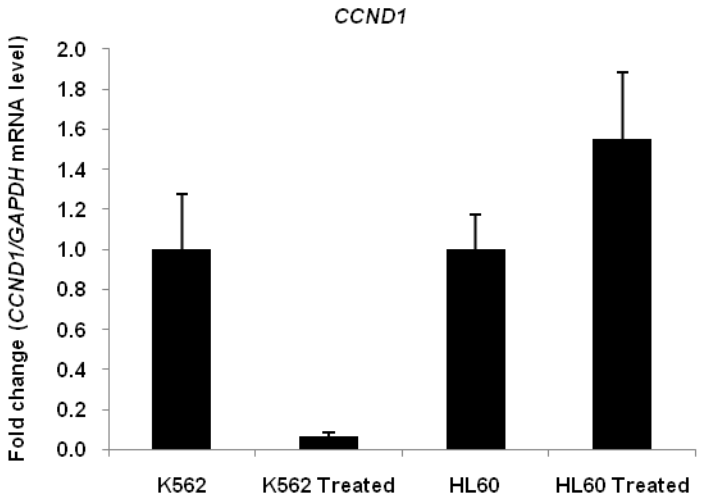

Fig. S8. Fold change of *CCND1* mRNA level following quantitative real-time PCR analysis in K562 and HL60 cells. Error bars represent standard deviation from three independent experiments.

Gene Symbol	Fold Change (K562 Treated vs. K562 Control) from microarray	Fold Change (K562 Treated vs. K562 Control) from real-time PCR
BCL2	-2.336	-6.029
PI3KCG	-2.292	-2.959
CCND1	-9.480	-14.379
TERT	1.028 (not significant)	-1.462

Table S2. Comparison of fold change obtained from microarray and real-time PCR.

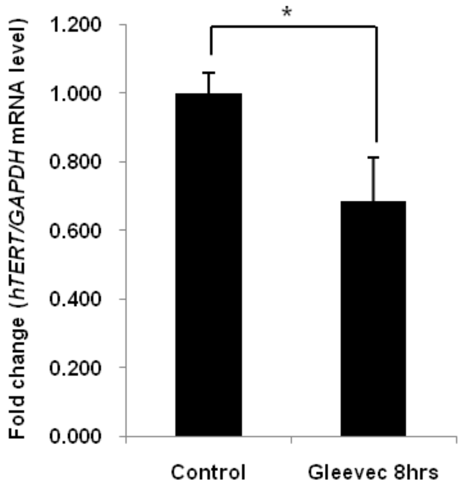

Fig. S9. Fold change of *hTERT* mRNA level following quantitative real-time PCR analysis in K562 cells under 1μM Gleevec for 8 hours. Error bars represent standard deviation from three independent experiments (* *p* value < 0.05).

A K562 Gleevec-treated group versus K562 control group

Description	Enrichment Score	Enrichment p-value	# genes in list, in group
Genes involved in Telomere Maintenance	7.39300	0.00062	12
Genes involved in Extension of Telomeres	5.00862	0.00668	5
Genes involved in Packaging of Telomere Ends	3.72121	0.02420	7

B K562 Gleevec-treated group versus K562 control group

Genes involved in Telomere Maintenance

Symbol	Definition	p-value (Drug Treatment vs. Control)	Fold-Change (Drug Treatment vs. Control)
HIST2H2AA3	Homo sapiens histone cluster 2, H2aa3 (HIST2H2AA3), mRNA.	0.00050	4.65402
HIST1H2AC	Homo sapiens histone cluster 1, H2ac (HIST1H2AC), mRNA.	0.00086	3.75160
RPA1	Homo sapiens replication protein A1, 70kDa (RPA1), mRNA.	0.00125	2.99833
HIST2H4B	Homo sapiens histone cluster 2, H4b (HIST2H4B), mRNA.	0.00100	2.94206
HIST1H2BJ	Homo sapiens histone cluster 1, H2bj (HIST1H2BJ), mRNA.	0.00064	2.91276
HIST1H2BD	Homo sapiens histone cluster 1, H2bd (HIST1H2BD), transcript variant 1, mRNA.	0.00089	2.86878
FEN1	Homo sapiens flap structure-specific endonuclease 1 (FEN1), mRNA.	0.00171	2.84345
TERF2	Homo sapiens telomeric repeat binding factor 2 (TERF2), mRNA.	0.00190	2.34197
HIST1H2BG	Homo sapiens histone cluster 1, H2bg (HIST1H2BG), mRNA.	0.00093	2.25438
POLD3	Homo sapiens polymerase (DNA-directed), delta 3, accessory subunit (POLD3), mRNA	0.01098	2.20271
RFC2	Homo sapiens replication factor C (activator 1) 2, 40kDa (RFC2), transcript variant 2, mRNA.	0.00726	2.13314
PRIM1	Homo sapiens primase, DNA, polypeptide 1 (49kDa) (PRIM1), mRNA.	0.00033	2.09809

Genes involved in Extension of Telomeres

Symbol	Definition	p-value (Drug Treatment vs. Control)	Fold-Change (Drug Treatment vs. Control)
RPA1	Homo sapiens replication protein A1, 70kDa (RPA1), mRNA.	0.00125	2.99833
FEN1	Homo sapiens flap structure-specific endonuclease 1 (FEN1), mRNA.	0.00171	2.84345
POLD3	Homo sapiens polymerase (DNA-directed), delta 3, accessory subunit (POLD3), mRNA	0.01098	2.20271
RFC2	Homo sapiens replication factor C (activator 1) 2, 40kDa (RFC2), transcript variant 2, mRNA.	0.00726	2.13314
PRIM1	Homo sapiens primase, DNA, polypeptide 1 (49kDa) (PRIM1), mRNA.	0.00033	2.09809

Genes involved in Packaging of Telomere Ends

Symbol	Definition	p-value (Drug Treatment vs. Control)	Fold-Change (Drug Treatment vs. Control)
HIST2H2AA3	Homo sapiens histone cluster 2, H2aa3 (HIST2H2AA3), mRNA.	0.00050	4.65402
HIST1H2AC	Homo sapiens histone cluster 1, H2ac (HIST1H2AC), mRNA.	0.00086	3.75160
HIST2H4B	Homo sapiens histone cluster 2, H4b (HIST2H4B), mRNA.	0.00100	2.94206
HIST1H2BJ	Homo sapiens histone cluster 1, H2bj (HIST1H2BJ), mRNA.	0.00064	2.91276
HIST1H2BD	Homo sapiens histone cluster 1, H2bd (HIST1H2BD), transcript variant 1, mRNA.	0.00089	2.86878
TERF2	Homo sapiens telomeric repeat binding factor 2 (TERF2), mRNA.	0.00190	2.34197
HIST1H2BG	Homo sapiens histone cluster 1, H2bg (HIST1H2BG), mRNA.	0.00093	2.25438

Table S3. Comparison between K562 Gleevec-treated group versus K562 control group. *A.* Gene set analysis showing enrichment of gene sets involved in telomere. These gene sets showed the number of genes that are upregulated in each group in K562 Gleevec-treated cells. *B.* List of upregulated genes relating to telomere maintenance, telomeres extension and telomere ends packaging in K562 Gleevec-treated cells.

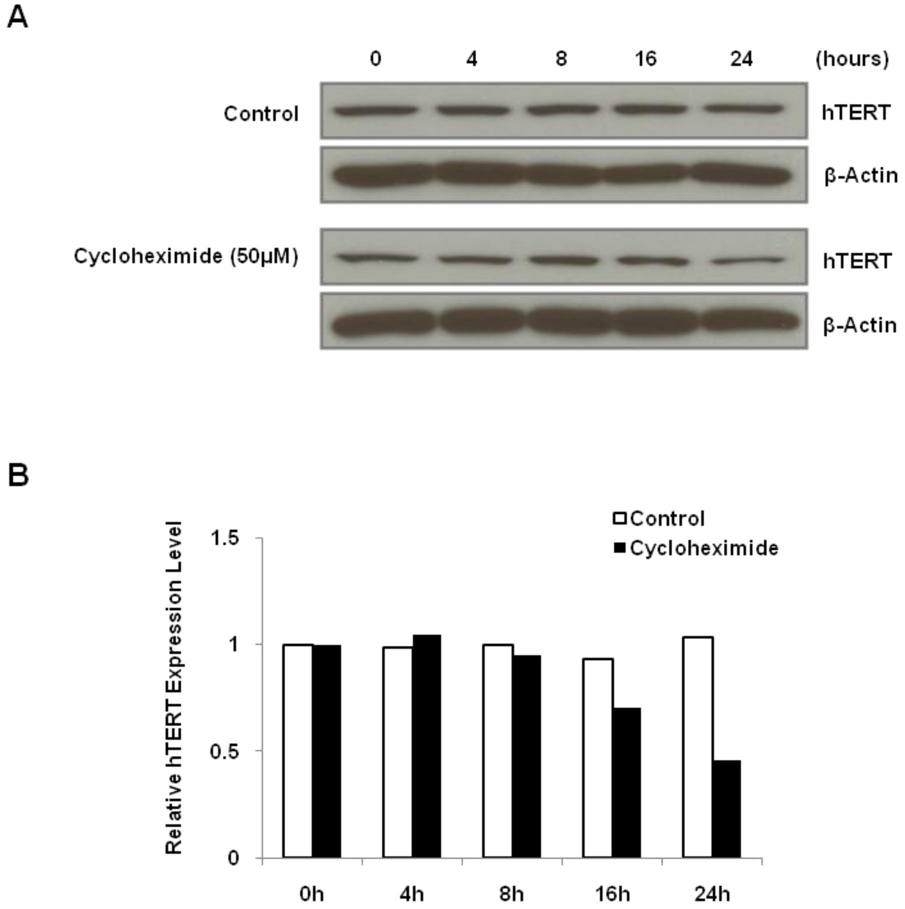

Fig. S10. Half-life of hTERT protein in K562 cells. *A.* Western blotting of hTERT under 50μM of Cycloheximide treatment for 24 hours. *B.* Quantitation of hTERT protein level showed half-life of hTERT protein in K562 cells is 24 hours approximately.

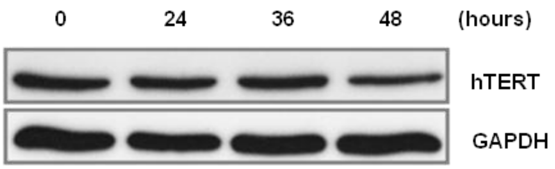

<u>Fig. S11.</u> Western blotting of hTERT under 1μM of Gleevec treatment for 24, 36 and 48 hours.

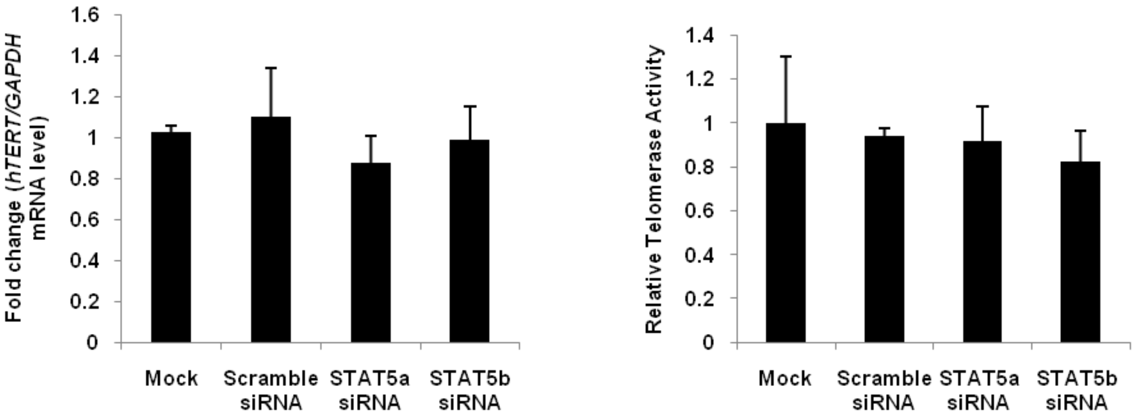

<u>Fig. S12.</u> After 72 hours of post-transfection, HL60 cells were collected and *hTERT* mRNA expression level and TA were measured by real-time PCR and quantitative telomerase assay, respectively.

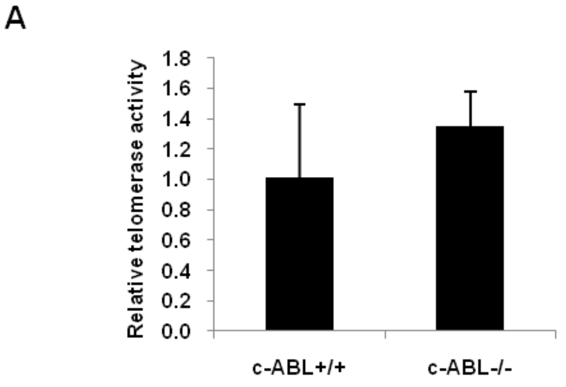

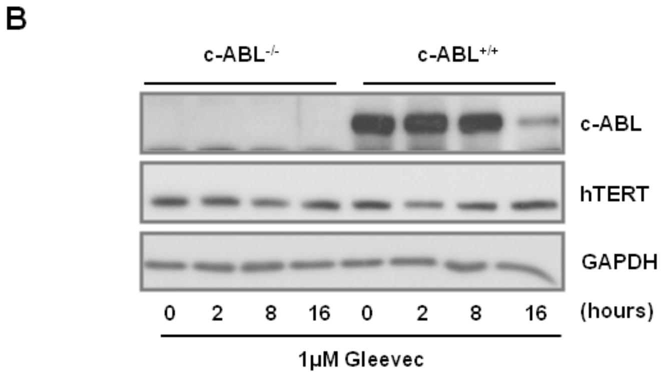

Fig. S13. TA and hTERT protein expression of c-ABL$^{-/-}$ and c-ABL$^{+/+}$ mouse embryonic fibroblasts (MEFs). *A.* c-ABL$^{-/-}$ MEFs showed no significant effect on TA by the c-ABL deficiency. Error bars represent standard deviation from three independent experiments. *B.* c-ABL$^{-/-}$ MEFs showed no significant effect on hTERT expression by c-ABL deficiency at protein level.
